# High-throughput binding affinity calculations at extreme scales

**DOI:** 10.1186/s12859-018-2506-6

**Published:** 2018-12-21

**Authors:** Jumana Dakka, Matteo Turilli, David W. Wright, Stefan J. Zasada, Vivek Balasubramanian, Shunzhou Wan, Peter V. Coveney, Shantenu Jha

**Affiliations:** 1 Department Electrical and Computer Engineering, Rutgers University, 94 Brett Road, Piscataway, NJ USA; 2 Centre for Computational Sciences, UCL, 20 Gordon Street, London, UK; 3 Institute for Advanced Computational Sciences, Stony Brook University, NY, USA, Lake Dr, Laufer Center, Stony Brook, NY USA; 4 Computational Science Initiative, Brookhaven National Laboratory, 98 Rochester St, Shirley, NY USA

## Abstract

**Background:**

Resistance to chemotherapy and molecularly targeted therapies is a major factor in limiting the effectiveness of cancer treatment. In many cases, resistance can be linked to genetic changes in target proteins, either pre-existing or evolutionarily selected during treatment. Key to overcoming this challenge is an understanding of the molecular determinants of drug binding. Using multi-stage pipelines of molecular simulations we can gain insights into the binding free energy and the residence time of a ligand, which can inform both stratified and personal treatment regimes and drug development. To support the scalable, adaptive and automated calculation of the binding free energy on high-performance computing resources, we introduce the High-throughput Binding Affinity Calculator (HTBAC). HTBAC uses a building block approach in order to attain both workflow flexibility and performance.

**Results:**

We demonstrate close to perfect weak scaling to hundreds of concurrent multi-stage binding affinity calculation pipelines. This permits a rapid time-to-solution that is essentially invariant of the calculation protocol, size of candidate ligands and number of ensemble simulations.

**Conclusions:**

As such, HTBAC advances the state of the art of binding affinity calculations and protocols. HTBAC provides the platform to enable scientists to study a wide range of cancer drugs and candidate ligands in order to support personalized clinical decision making based on genome sequencing and drug discovery.

## Background

In recent years, chemotherapy based on targeted kinase inhibitors (TKIs) has played an increasingly prominent role in the treatment of cancer. TKIs have been developed to selectively inhibit kinases involved in the signaling pathways that control growth and proliferation, which often become dysregulated in cancers. This targeting of specific cancers reduces the risk of damage to healthy cells and increases treatment success. Currently, 35 FDA-approved small molecule TKIs are in clinical use, and they represent a significant fraction of the $37 billion U.S. market for oncology drugs [1, 2]. Imatinib, the first of these of drugs, is partially credited for doubling survivorship rates in certain cancers [2, 3].

**Fig. 1:**
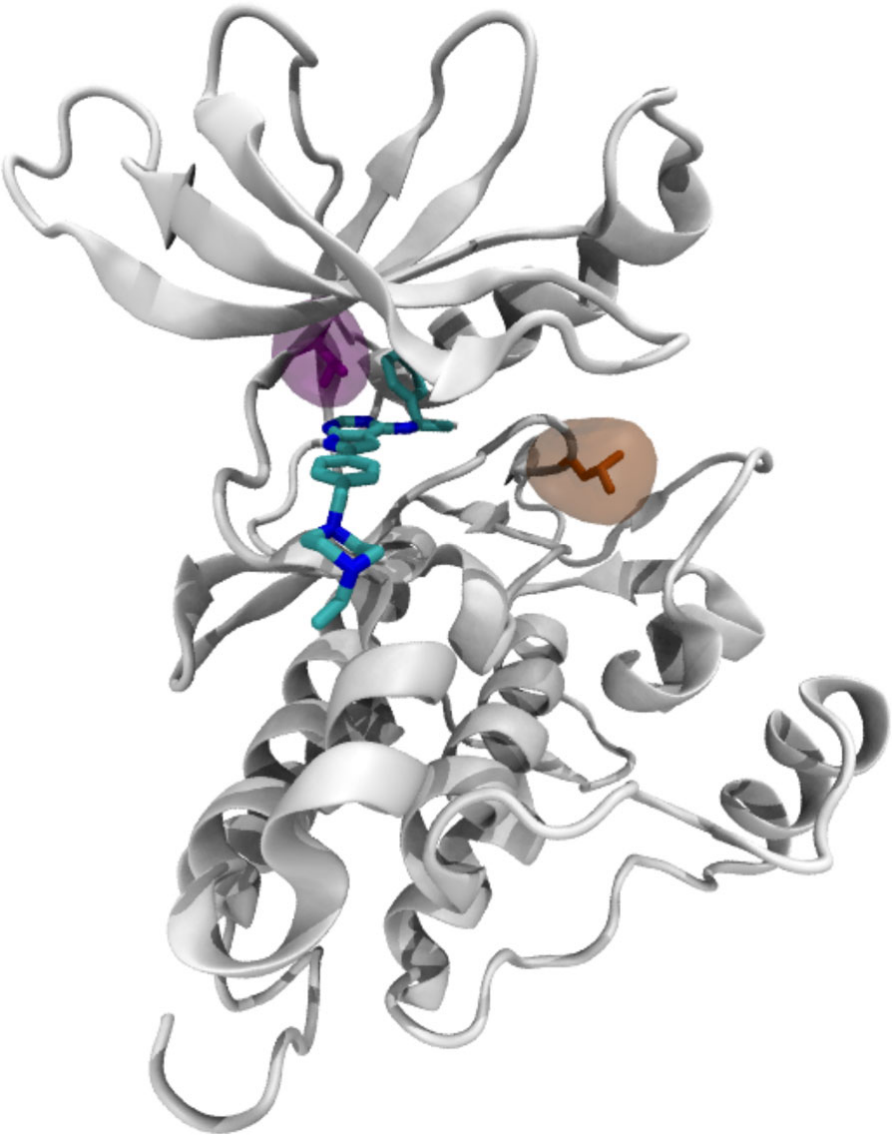
EGFR Structure. Cartoon representation of the EGFR kinase bound to the inhibitor AEE788 shown in chemical representation (based on PDB:2J6M). Two residues implicated in modulating drug efficacy are highlights; in pink T790 and in orange L858. Mutations to either of these residues significantly alter the sensitivity to TKIs

Unfortunately, the development of resistance to these drugs limits the amount of time that patients can derive benefits from their treatment. Resistance to therapeutics is responsible for more than 90% of deaths in patients with metastatic cancer [4]. While drug resistance can emerge via multiple mechanisms, small changes to the chemical composition of the therapeutic target (known as mutations) control treatment sensitivity and drive drug resistance in many patients (see Fig. 1). In some commonly targeted kinases such as Abl, these changes account for as many as 90% of treatment failure [5].

There are two major strategies for countering the threat to treatment efficacy posed by resistance: tailoring the drug regimen received by a patient according to the mutations present in their particular cancer, and developing more advanced therapies that retain potency for known resistance mutations. In both cases, future developments require insight into the molecular changes produced by mutations, as well as ways to predict their impact on drug binding on a timescale much shorter than is typically experimentally feasible. This represents a grand challenge for computational approaches.

The rapidly decreasing cost of next-generation sequencing has led many cancer centers to begin deep sequencing of patient tumors to identify the genetic alterations driving individual cancers. The ultimate goal is to make individualized therapeutic decisions based upon these data—an approach termed *precision cancer therapy*. While several common (recurrent) mutations have been cataloged for their ability to induce resistance or for their susceptibility to particular kinase inhibitors, the vast majority of clinically observed mutations are rare [6, 7]. Essentially, this ensures that it will be impossible to make therapeutic decisions about the majority of individual patient tumors by using catalog-building alone.

Fortunately, concurrent improvements in computational power and algorithm design are enabling the use of molecular simulations to reliably quantify differences in binding strength. This provides the opportunity to use advances in molecular simulations to supplement existing inductive decision support systems with deductive predictive modeling and drug ranking [8, 9]. Where existing systems based on statistical inference are inherently limited in their range of applicability by the existence of data from previous similar cases, the addition of modeling allows evidence based decision making even in the absence of direct past experience.

The same molecular simulation technologies that can be employed to investigate the origins of drug resistance can also be used to design new therapeutics. Creating simulation protocols which have well defined uncertainty and produce statistically meaningful results represents a significant computational challenge. Furthermore, it is highly likely that differences among investigated systems will demand different protocols as studies progress. For example, drug design programmes often require the rapid screening of thousands of candidate compounds to filter out the worst binders before using more sensitive methods to refine the structure. Not all changes induced in protein shape or behavior are local to the drug binding site and, in some cases, simulation protocols will need to adjust to account for complex interactions between drugs and their targets within individual studies.

Recent work that used molecular simulations to provide input to machine learning models [10] required simulations of 87 compounds even if they were designed merely to distinguish the highly active from weak inhibitors of the ERK2 kinase. If we wish to build on such studies to help inform later stages of the drug discovery pipeline, in which much more subtle alterations are involved, it is likely a much larger number of simulations will be required. This is before we begin to consider the influence of mutations or the selectivity of drugs to the more than 500 different genes in the human kinome [11].

For molecular simulations to make the necessary impact, the dual challenge of scale (thousands of concurrent multi-stage pipelines) and sophistication (adaptive selection of binding affinity protocols based upon statistical errors and uncertainty) will need to be tackled. Tools that facilitate the scalable and automated computation of varied binding free energy calculations on high-performance computing resources are necessary. To achieve that goal, we introduce the High-throughput Binding Affinity Calculator (HTBAC). HTBAC applies recent advances in workflow system building blocks to the accurate calculation of binding affinities, executing hundreds of concurrent calculations on a leadership class machine [12]. This permits the rapid time-to-solution that is essentially invariant of the size of candidate ligands as well as the type and number of protocols concurrently employed.

In the next section, we provide details of ensemble molecular dynamics approach and its advantages over the single trajectory approach. We also introduce the ESMACS and related protocols to compute binding affinities using ensemble-based approaches. In “Methods” section, we discuss the computational challenges associated with the scalable execution of multiple, and possibly concurrently executing protocols. Also, in “Methods” section, we introduce RADICAL-Cybertools—a suite of building blocks to address the computational challenges—and describe how they are used by HTBAC to manage the execution of binding affinity calculations at extreme scales. Experiments to characterize the performance overheads of RADICAL-Cybertools and the weak scaling properties of the HTBAC implementation of the ESMACS protocol on the Blue Waters supercomputer are discussed in “Results” section. We conclude with a discussion of the impact of HTBAC, implication for binding affinity calculations and near-term future work.

## Methods

The strength of drug binding is determined by a thermodynamic property known as the binding free energy (or binding affinity). One promising technology for estimating binding free energies and the influence of protein composition on them is molecular dynamics (MD) [13]. Our previous work [14, 15] has demonstrated that running multiple MD simulations based on the same system and varying only in initial velocities offers a highly efficient method of obtaining accurate and reproducible estimates of the binding affinity. We term this approach ensemble molecular dynamics, “ensemble” here referring to the set of individual (replica) simulations conducted for the same physical system. In this Section we discuss the advantages to this approach.

### Ensemble molecular dynamics simulations

Atomistically detailed models of the drug and target protein can be used as the starting point for MD simulations to study the influence of mutations on drug binding. The chemistry of the system is encoded in what is known as a potential [16]. In the parameterization of the models, each atom is assigned a mass and a charge, with the chemical bonds between them modeled as springs with varying stiffness. Using Newtonian mechanics the dynamics of the protein and drug can be followed and, using the principles of statistical mechanics, estimates of thermodynamic properties obtained from simulations of single particles.

The potentials used in the simulations are completely under the control of the user. This allows the user to manipulate the system in ways which would not be possible in experiments. A particularly powerful example of this are the so called “alchemical” simulations in which the potential used in the simulation changes, from representing a particular starting system into one describing a related target system during execution. This allows for the calculation of free energy differences between the two systems, such as those induced by a protein mutation.

MD simulations can reveal how interactions change as a result of mutations, and account for the molecular basis of drug efficacy. This understanding can form the basis for structure-based drug design as well as helping to target existing therapies based on protein composition. However, correctly capturing the system behavior poses at least two major challenges: The approximations made in the potential must be accurate enough representations of the real system chemistry; and sufficient sampling of phase space is also required.

In order for MD simulations to be used as part of clinical decision support systems, it is necessary that results can be obtained in a timely fashion. Typically, interventions are made on a timescale of a few days or, at most, a week. The necessity for rapid turn around times places additional demands on simulation protocols which need to be optimized to gain results with a short turn around time. Further to the scientific and practical considerations outlined above, it is vital that reliable uncertainty estimates are provided alongside all quantitative results for simulations to provide actionable predictions.

We have developed a number of free energy calculation protocols based on the use of ensemble molecular dynamics simulations with the aim of meeting these requirements [14, 17–19]. Basing these computations on the direct calculation of ensemble averages facilitates the determination of statistically meaningful results along with complete control of errors. The use of the ensemble approaches however, necessitates the use of middleware to provide reliable coordination and distribution mechanisms with low performance overheads.

### Protocols for binding affinity calculations

We have demonstrated the lack of reproducibility of single trajectory approaches in both HIV-1 protease and MHC systems, with calculations for the same protein-ligand combination, with identical initial structure and force field, shown to produce binding affinities varying by up to 12 kcal mol ^−1^ for small ligands (flexible ligands can vary even more) [14, 20, 21]. Indeed, our work has revealed how completely unreliable single simulation based approaches are.

Our work using ensemble simulations have also reliably produced results in agreement with previously published experimental findings [14, 15, 18, 19, 21, 22], and correctly predicted the results of experimental studies performed by colleagues in collaboration [23]. While the accuracy of force fields could be a source of error, we know from our work to date that the very large fluctuations in trajectory-based calculations account for the lion’s share of the variance (hence also uncertainty) of the results. For example, our reproduction of ligand simulations from [18] in a more recent version of the Amber forcefield (ff14 as opposed to ff99SBildn) produced average values within 0.5 kcal mol ^−1^, less than a 20th of the range observed in the original ensemble.

We designed two free energy calculation protocols with the demands of clinical decision support and drug design applications in mind: ESMACS (enhanced sampling of molecular dynamics with approximation of continuum solvent) [18] and TIES (thermodynamic integration with enhanced sampling) [22]. The former protocol is based on variants of the molecular mechanics Poisson-Boltzmann surface area (MMPBSA) end-point method, the latter on the ‘alchemical’ thermodynamic integration (TI) approach. A wide variety of free energy methods are available, ranging from very fast molecular docking methods to rigorous but expensive absolute binding free energy methods [24]. The methods examined here - MMPBSA based approximate absolute and TI based rigorous relative calculations - exist between these two extremes. It should be noted that there are many methods for analysing the results of relative free energy computations. Here we use the simple TI formalism, the relative performance of more complex schemes, such as those based on Bennett’s acceptance ratio, has been seen to be highly system dependent [25–27].

In both ESMACS and TIES, ensembles of MD simulations are employed to perform averaging and to obtain tight control of error bounds in our estimates. In addition, the ability to run replica simulations concurrently means that, as long as sufficient compute resources are available, turn around times can be significantly reduced compared to the generation of single long trajectories. The common philosophy behind the two protocols entails similar middleware requirements: In this work we focus on the ESMACS protocol but all results are applicable also to TIES. In [12] we focus on the performance of HTBAC using the TIES protocol.

Each replica within the ESMACS protocol consists of a sequence of simulation stages followed by post production analysis. Generally, an ESMACS replica will contain between 3 and 12 equilibration simulation stages followed by a production MD run, all of which are conducted in the NAMD package [28]. The first stage is system minimization, the following stages involve the gradual release of positional constraints upon the structure and the heating to a physiologically realistic temperature. Upon completion of the MD simulation, free energy computation (via MMPBSA and potentially normal mode analysis) is performed using AmberTools [29, 30].

The ESMACS protocol is highly customizable. Both the number of simulation replicas in the ensemble and the lengths of their runs can be varied to obtain optimal performance for any given system. Using replicas that only vary in the initial velocities assigned to the atoms of the system we have defined a standard protocol which prescribes a 25 replica ensemble, each run consisting of 2 ns of equilibration and 4 ns of production simulation. Our protocol has produced bootstrap errors of below 1.5 kcal mol ^−1^ (despite replica values varying by more than 10 kcal mol ^−1^) for a varied range of systems including small molecules bound to kinases and more flexible peptide ligands binding to MHC proteins [18, 20, 21]. In these systems, the error we obtained more than halves between ensembles of 5 and 25 replicas but increases in ensemble size have generally produced only small improvements. More generally though, there may be cases where it is important to increase the sampling of phase space either through expanding the ensemble or by considering multiple initial configurations. For example, systems such as kinases where flexible loops impact the binding site interactions may require the use of much greater levels of simulation to obtain correctly converged results.

The ESMACS protocol can also be extended to account for adaptation energies involved in altering the conformation of the protein or ligand during binding. Almost all MMPBSA studies in the literature use the so-called 1-trajectory method, in which the energies of protein-inhibitor complexes, receptor proteins and ligands are extracted from the MD trajectories of the complexes alone. The ESMACS protocol can additionally use separate ligand and receptor trajectories to account for adaptation energies, providing further motivation to deploy the protocol via flexible and scalable middleware.

### Benchmark kinase system

A common target of kinase inhibitors is the epidermal growth factor receptor (EGFR) which regulates important cellular processes including proliferation, differentiation and apoptosis. EGFR is frequently over expressed in a range of cancers, and is associated with disease progression and treatment. Clinical studies have shown that EGFR mutations confer tumor sensitivity to tyrosine kinase inhibitors in patients with non-small-cell lung cancer (examples shown in Fig. 1) The tyrosine kinase domain of EGFR contains 288 residues, the full simulation system including solvent and the AEE788 inhibitor contains approximately 50 thousand atoms. The well established AMBER ff99SBildn and GAFF force fields [31, 32] were used to parameterize the system for this work.

### Automated binding affinity calculations

The implementation of any physically realistic molecular simulation has always been an involved and multistage process, often requiring the scientist to overcome a large manual overhead in the construction, preparation, and execution protocols necessary to complete a set of simulations as well as to invoke various analysis protocols for determining desired properties post-production.

Several tools have been been developed to automate MD workflows for the rapid, accurate and reproducible computation of binding free energies of small molecules to their target proteins. For example, BAC [17] is a partially automated workflow system which comprises (a) model building (including incorporation of mutations into patient specific protein models), (b) running ensembles of MD simulations using a range of free energy techniques and (c) statistical analysis. In “ESMACS” section, we decribed how we have enhanced BAC using the RADICAL-Cybertools to produce (HTBAC).

### Computational challenges at scale

High-performance computing (HPC) environments were designed to primarily support the execution of single simulations. Current HPC platforms enable the strong and weak scaling of single tasks (hitherto mostly simulations), with limited software and systems support for the concurrent execution of multiple heterogeneous tasks as part of a single application (or workflow). As the nature of scientific inquiry and the applications to support that inquiry evolve, there is a critical need to support the scalable and concurrent execution of a large number of heterogeneous tasks.

Sets of tasks with dependencies that determine the order of their execution are usually referred to as “workflows”. Often times, the structure of the task dependencies is simple and adheres to discernible patterns, even though the individual tasks and their duration are non-trivially distinct. Put together, it is a challenge to support the scalable execution of workflows on HPC resources due to the existing software ecosystem and runtime systems typically found.

Many workflow systems have emerged in response to the aforementioned problem. Each workflow system has its strengths and unique capability, however each system typically introduces its problems and challenges. In spite of the many successes of workflow systems, there is a perceived high barrier-to-entry, integration overhead and limited flexibility.

Interestingly, many commonly used workflow systems in high-performance and distributed computing emerged from an era when the software landscape supporting distributed computing was fragile, missing features and services. Not surprisingly, initial workflow systems had a monolithic design that included the end-to-end capabilities needed to execute workflows on heterogeneous and distributed cyberinfrastructures. Further, these workflow systems were typically designed by a set of specialists to support large “big science” projects such as those carried out at the LHC [33] or LIGO [34]. The fact that the same workflow would be used by thousands of scientists over many years justified, if not amortized, the large overhead of integrating application workflows with monolithic workflow systems. This influenced the design and implementation of interfaces and programming models.

Executing biomolecular applications on HPC systems require specific knowledge of resource, data, and execution management. Several middleware frameworks [35] have been developed to abstract some of these details from the user. For example, gSOAP [36] enables web services for HPC applications while Ninf-G [37] and OmniRPC [38] support distributed programming via a client/server architecture. These solutions provide methods to launch application tasks on remote machines but leave the details of task scheduling, resource and data management to the user. On the other hand, domain-specific workflows provide a customized interface to the domain scientist, but require users to manage resource selection and setup the execution environments on the HPC system.

However as the nature, number and usage of workflows has evolved so have the requirements: scale remains important but only when delivered with the ability to prototype quickly and flexibly. Furthermore, there are also new performance requirements that arise from the need to support concurrent execution of heterogeneous tasks. For example, when executing multiple homogeneous pipelines of heterogeneous tasks, for reasons of efficient resource utilization there is a need to ensure that the individual pipelines have similar execution times. The pipeline-to-pipeline fluctuation must be minimal while also managing the task-to-task runtime fluctuation across concurrently executing pipelines.

Thus the flexible execution of heterogeneous ensembles MD simulations face both system software and middleware challenges: existing system software that is typically designed to support the execution of single large simulations on the one hand, and workflow systems that are designed to support specific use cases or ‘locked-in’ end-to-end executions. In the next sections, we discuss the design and implementation of the RADICAL-Cybertools, a set of software building blocks that can be composed to design, implement and execute domain specific workflows rapidly and at scale.

### RADICAL-Cybertools

We have designed RADICAL-Cybertools (RCT) to be functionally delineated middleware building blocks and to address some of the challenges in developing and executing workflows on HPC platforms. HTBAC uses two RCT components, mainly the Ensemble Toolkit (EnTK) and RADICAL-Pilot (RP). EnTK provides the ability to create and execute ensemble-based workflows/applications with complex coordination and communication but without the need for explicit resource management. EnTK uses RP as a runtime system which provides resource management and task execution capabilities.

RCT eschew the concept of a monolithic workflow systems and uses “building blocks”. RCT provide scalable implementations of building blocks in Python that are used to support dozens of scientific applications on high-performance and distributed systems [39–43]. In this Section we discuss details of RP, EnTK and HTBAC, understanding how these components have been used to support the flexible and scalable execution of pipelines.

### RADICAL-Pilot

The scalable execution of applications with large ensembles of tasks is challenging. Traditionally, two methods are used to execute multiple tasks on a resource: each task is scheduled as an individual job, or message-passing interface (MPI) capabilities are used to execute multiple tasks as part of a single job. The former method suffers from unpredictable queue time: each task independently awaits in the resource’s queue to be scheduled. The latter method relies on the fault tolerance of MPI, and is suitable to execute tasks that are homogeneous and have no interdependencies.

The Pilot abstraction [44] solves these issues: The pilot abstraction: (i) uses a placeholder job without any tasks assigned to it, so as to acquire resources via the local resource management system (LRMS); and, (ii) decouples the initial resource acquisition from task-to-resource assignment. Once the pilot (container-job) is scheduled via the LRMS, it can pull computational tasks for execution. This functionality allows all the computational tasks to be executed directly on the resources, without being queued via the LRMS. The pilot abstraction thus supports the requirements of task-level parallelism and high-throughput as needed by science drivers, without affecting or circumventing the queue policies of HPC resources.

RADICAL-Pilot is an implementation of the pilot abstraction, engineered to support scalable and efficient launching of heterogeneous tasks across different platforms.

### Ensemble Toolkit

An ensemble-based application is a **workflow**, i.e. tasks with dependencies that determine the order of their execution. Subsets of these tasks can be **workloads**, i.e., tasks whose dependencies have been satisfied at a particular time and may be executed concurrently. Ensemble-based application vary in the type of coupling between tasks, the frequency and volume of information exchanged between these tasks, and the executable of each task. This type of applications requires specific coordination, orchestration and execution protocols, posing both domain-specific and engineering challenges.

Ensemble Toolkit (EnTK), the topmost layer of RCT, simplifies the process of creating and executing ensemble-based applications with complex coordination and communication requirements. EnTK decouples the description of ensemble-based applications from their execution by separating three orders of concern: specification of task and resource requirements; resource selection and acquisition; and task execution management. Domain scientists retain full control of the implementation of their algorithms, programming ensemble-based applications by describing what, when and where should be executed. EnTK uses a runtime system, like RADICAL-Pilot, to acquire the resources needed by applications to manage task execution.

**Fig. 2:**
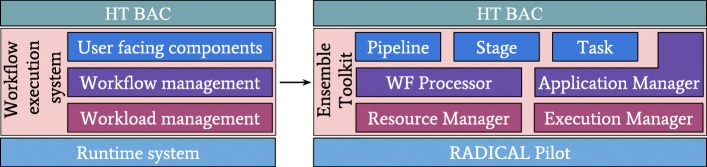
EnTK Overview. Ensemble Toolkit overview showing how the abstract workflow execution system is mapped to specific components exposed to the users and components internal to the toolkit

EnTK enables the creation of ensemble-based applications by exposing an application programming interface (API) with four components: **Application Manager**, **Pipeline**, **Stage** and **Task**. Users describe ensembles in terms of pipelines, stages and tasks, and pass this description to an instance of Application Manager, specifying what resource to use for executing the application (see Fig. 2).

The Task component is used to encapsulate an executable and its software environment and data dependencies. The Stage component contains a set of tasks without mutual dependencies and that can therefore be executed concurrently. The Pipeline component is used to describe a sequence of stages, i.e., sets of tasks that need to be executed sequentially, not concurrently.

The use of the Task, Stage, and Pipeline components, implemented as set and list data structures, avoids the need to express explicitly relationship among tasks. These relationships are insured by design, depending on the formal properties of the lists and sets used to partition tasks into stages and group stages into pipelines. Further, EnTK enables an explicit definition of pre and post conditions on the execution of tasks, enabling a fine grained adaptivity, both a local and global level. Conveniently, this does not require the codification of a directed acyclic graph (DAG), a process that imposes a rigid representation model on the domain scientists [45].

The **Application Manager** component of EnTK enables users to specify target resources for the execution of the ensemble-based application. This includes properties like walltime, number of nodes and credentials for resource access. Users can also define execution setup parameters such as the number of processes or messaging queues that should be used by EnTK. This allows to size and tune the performance of EnTK, depending on the number of tasks, stages and pipelines, but also on the resources available to the toolkit.

The Application Manager along with the **WF Processor** is responsible for the transformation of the application workflow into workloads, i.e., set of tasks, that can be submitted to the indicated resources for execution. Internally, the **Resource Manager** and **Execution Manager** components enable the acquisition of resources and the management of execution of these workloads (see Fig. 2).

### ESMACS

HTBAC captures the workflow logic of binding affinity calculation protocols using existing RCT tools. Initially, we designed HTBAC to implement a single binding affinity protocol, using the EnTK programming model to express the application logic. Here, we exclusively focus on ESMACS to capture the workflow logic and isolate the performance of a single protocol instance, by describing the ESMACS protocol directly with the user-facing API of EnTK. HTBAC has been extended as a Python library that enables the selection of multiple protocol instances of ESMACS and TIES [12]. To this end, we establish a distinction between HTBAC, the Python library, and the ESMACS implementation using EnTK.

A simulation pipeline is a defined sequence of simulation stages for a given physical system. In the ESMACS protocol, these simulation pipelines are replicated, where replicas differ only by their parameter configurations, namely initial velocities, which are randomly generated and assigned by NAMD at the start of execution. A simulation pipeline in the ESMACS protocol has 7 stages: the first, second and last stages perform staging of the input/output data, the middle stages indicate simulation tasks. A task is appended to a stage and stages are appended to a pipeline to maintain temporal order during execution.

**Fig. 3:**
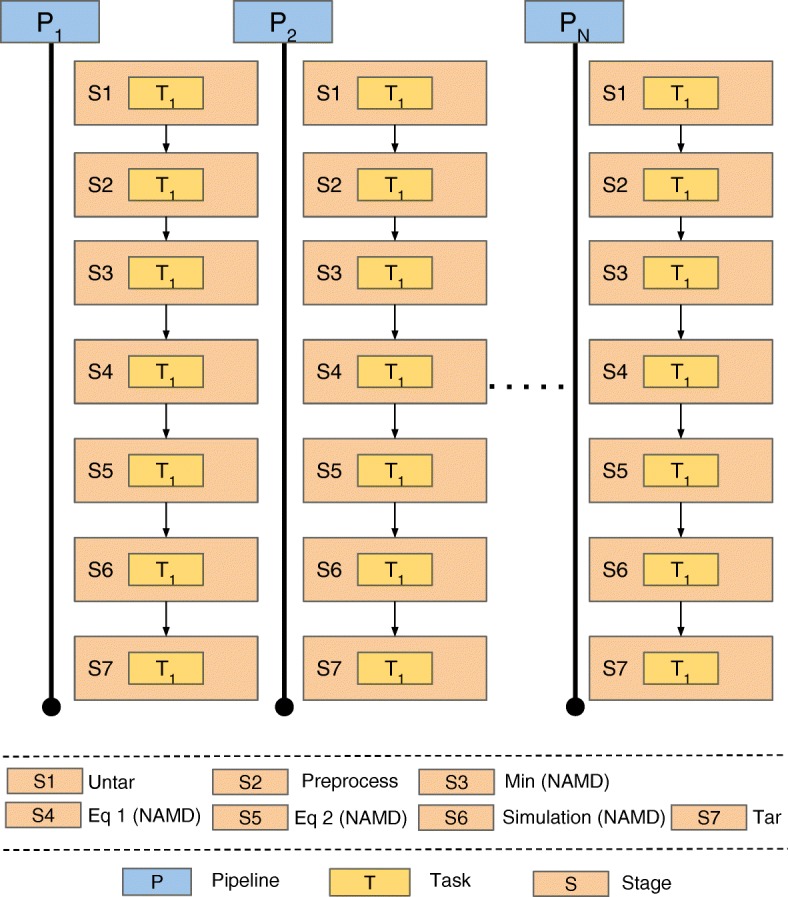
ESMACS Implementation using EnTK. ESMACS protocol implemented as an ensemble application, encoded using the EnTK API. A protocol represents a physical system and is encoded as a set of independent pipelines. Each pipeline maps to a single replica. ESMACS consists of 25 replicas. Stages within a pipeline are executed sequentially. Each stage contain a single task performing unique functions, as required by the protocol. Stages S3–S6 contain molecular dynamics simulation tasks executed with NAMD

Each simulation pipeline replica maps to an independent EnTK pipeline. Each pipeline consists of a sequence of stages, and each stage consists of a single task that performs unique functions, including pre-processing and molecular dynamics simulations. Figure 3 shows how pipelines, stages and tasks are organized for a single ESMACS protocol instance. A task is composed of a set of attributes that define parameters like the location of input files, the number of simulations and the MD engine(s) used to launch those simulations.

**Fig. 4:**
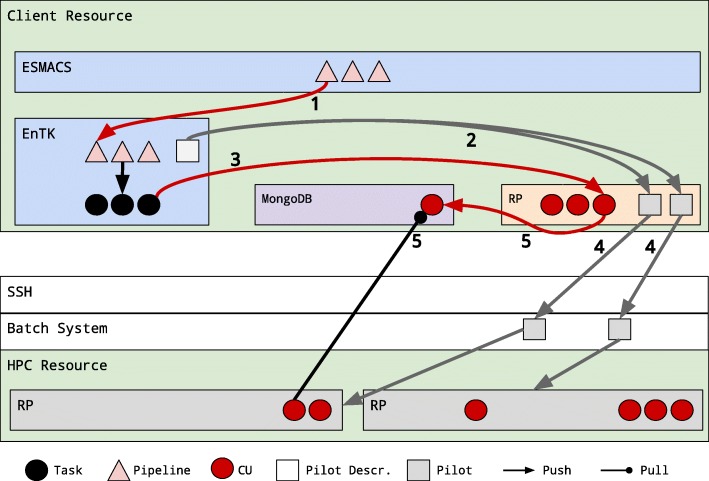
ESMACS-EnTK-RP Integration. Integration between ESMACS and EnTK. Numbers indicate the temporal sequence of execution. The database (DB) of RADICAL-Pilot (RP) can be deployed on any host reachable from the resources. RP pushes compute units (CU) to DB and pulls them for execution

Figure 4 shows how the ESMACS protocol integrates with EnTK. EnTK converts the set of pipelines into a set of tasks called compute unit descriptions and submits them to RP. In addition, EnTK provides methods for the user to specify a resource request including walltime, cores, queue, and user credentials. EnTK converts this resource request into a pilot that RP submits to a HPC machine. Once the pilot becomes active, it pulls compute unit descriptions in bulk from a database, executing them on the pilot resources.

## Results

Before embarking on a computational campaign that will consume 150M core hours on the NCSA Blue Waters machine, we studied the scalability of HTBAC so as to determine optimal workflow sizing and resource utilization for the ESMACS protocol. The goal is twofold: (1) understanding the invariance of HTBAC execution time for a single protocol instance over the number of workflow pipelines executed; and (2) studying how the performance of EnTK and RP varies in relation to the size of workflow.

### Experiment design

We designed two experiments to measure weak scalability properties using the ESMACS protocol when executing an increasing number of concurrent pipelines. According to the use case described in “ESMACS” section, each pipeline consists of seven stages, each stage with a single task. EnTK manages the queuing of the tasks in accordance with the order and concurrency mandated by stages and pipelines: For each pipeline, each stage is executed sequentially while pipelines are executed concurrently.

Experiment 1 measures the baseline behavior of EnTK and RP with the workflow of the ESMACS protocol and a null workload (/bin/sleep 0). The goal is to isolate the overheads of EnTK and RP from the specifics of the executables of the workflow and the overheads of the resources. The null workload does not require data staging, I/O on both memory and disk, or communication over network.

Experiment 2 replicates the design of Experiment 1 but it executes the workflow of the ESMACS protocol with the actual simulation and data for the EGFR kinase workload. The comparison between the two experiments enables performance analysis of EnTK and RP to understand whether and how the size of the executed workflow affects its overheads. Further, Experiment 2 shows also whether HTBAC execution time is sensitive to the number of concurrent pipelines executed.

Both experiments measure the weak scalability properties of a single ESMACS protocol instance, executed with RCT. This means that the ratio between the number of pipelines and cores is kept constant by design. While an investigation of strong scalability would contribute to a better understanding of the behavior of a single ESMACS protocol instance, it is of limited interest for the current use case. The driving goal of this study is to increase throughput by a means of concurrency of pipelines, not in the number of sequential executions per core. This is a driving motivation to target large HPC machines instead of so-called high-throughput computing (HTC) infrastructures.

### Experiment setup

We perform both Experiment 1 and 2 on NCSA’s Blue Waters—a 13.3 petaFLOPS Cray, with 32 Interlago cores/50 GB RAM per node, Cray Gemini, Lustre shared file system. Currently, we exclusively use CPUs on Blue Waters as GPUs are not required by our use case.

We perform our experiments from a virtual machine, which maintains a stable network connection. This helps to simulate the conditions in which the experimental campaign will be performed by the research group at UCL, where users run experiments from local machines.

To this end, we perform our experiments from a virtual machine. This helps to simulate the conditions in which the experimental campaign will be performed from a local machine. To allow this, RCT support gsissh for authentication and authorization. The virtual machine serves as the client from which the user provides the description of the experiments including the physical systems, and resource requirements. Once the experiment is submitted, RCT establish a gsissh connection from the virtual machine to the remote resource i.e. Blue Waters and maintain a tunnel for the duration of the experiment.

Table 1 shows the setup for Experiment 1 and 2. The ESMACS protocol is executed with up to 25 concurrent but independent pipelines and therefore their concurrent execution does not entail communication overhead. While we show that EnTK can support concurrent execution of 128 pipelines, ESMACS only requires 25 concurrent pipelines. We demonstrate scales beyond 25 pipelines to address the resource requirements of more computationally demanding free energy protocols. Furthermore, the EGFR kinase studies can benefit from greater concurrency because potential HTBAC users may wish to extend their protocols beyond the current scale of ESMACS by executing more than one workload in a single run. Consistently, our experiments push the boundaries of current scale by executing 8, 16, 32, 64 and 128 concurrent pipelines.

**Table 1:** Experiment 1 executes the 7 stages of the ESMACS protocol with a null workload; Experiment 2 uses the actual MD workload of the ESMACS protocol

Experiment ID	Protocol	Workload	# Trials	# Pipelines	# Stages	# Tasks	# Cores per pilot
1	ESMACS	Null workload	2	8, 16, 32, 64, 128	7	7	64, 128, 256, 512, 1024
2	ESMACS	EGFR kinase Workload	2	8, 16, 32, 64, 128	7	7	64, 128, 256, 512, 1024

ESMACS protocol with EGFR kinase workload: (1) Untar configuration files; (2) Preprep; (3) Minimize with decreasing restraints; (4) Equilibration: NVT simulation at 50K, with restraints; (5) Equilibration: NPT simulation at 300K, with decreasing restraints; (6) Equilibration: NPT at 300k, no constraints; (7) Tarball output files

EnTK uses RP to acquire resources via a single pilot. The size of the pilot is contingent upon characterization of performance, in this case, weak scalability. Accordingly, we request the maximum number of cores required by the workload as the number of cores in a pilot. We use between 64 and 1024 cores in Experiment 2 as the NAMD executable used in stages 3, 4, 5, and 6 requires 8 cores. Stages 1, 2 and 7 require instead 1 core. The null workload of Experiment 1 requires only 1 core per stage but we request the same number of cores as for Experiment 2 to be able to compare the overheads of both EnTK and RP across experiments.

All experiments use EnTK version 0.4.7 and RP version 0.42. The MD engine used is NAMD-MPI. The equilibration tasks of stage 4 and 6 are assigned 5000 timesteps while the task of stage 5 requires 55000 timesteps. We ran two trials of both the null and MD workload at each pipeline configurations.

### Results

**Fig. 5:**
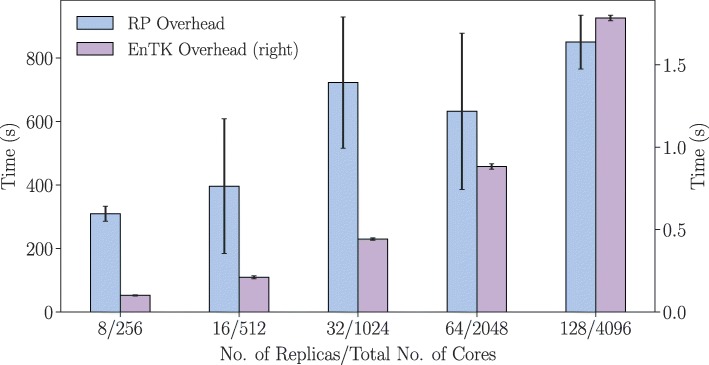
Null Workload Overheads. Overheads of Ensemble Toolkit (EnTK) and RADICAL-Pilot (RP) when executing HTBAC using a null workload. We plot the baseline EnTK/RP overheads without the application workload across two trials per pipeline configuration

First we characterize the overhead of EnTK and RP in the null workload, where we isolate the overhead introduced by the two systems (Fig. 5). We see a (slightly) superlinear increase of EnTK overhead, between 0.1 and 1.8 s. This overhead depends on the number of tasks that need to be translated in-memory from a Python object to a CU description. As such, it is expected to grow proportionally to the number of tasks, barring some competition of resources.

RP overhead is also, on average, superlinear but with a much greater variance. This variance is due to mainly two factors: Network latency and filesystem latency on the HPC resource. EnTK submits CU descriptions to the MongoDB used by RP, and the RP pilot pulls these descriptions from the same database. As described in “Experiment setup” section, this pull operation occurs over a wide area network, introducing varying amounts of latency. Further, RP writes and reads the CU descriptions multiple times to and from the shared filesystem of the HPC machine. Together, these two factors introduce delays in the scheduling of the CUs.

**Fig. 6:**
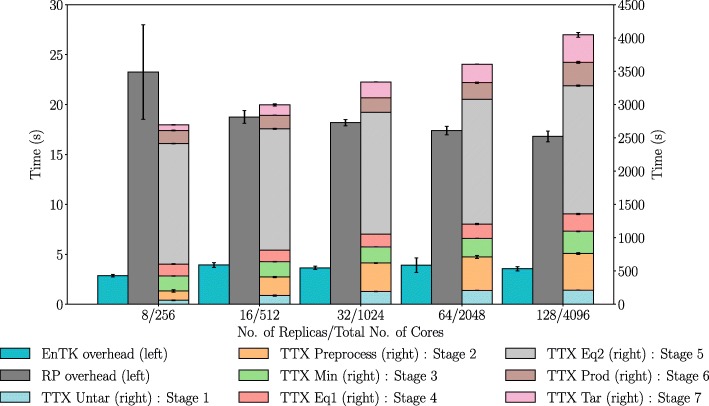
Weak Scaling ESMACS Protocol. similar EnTK/RP overhead behavior as with the null workload with higher values as the number of pipelines increases. We show a breakdown of TTX of each stage (Stage 1–7). Across pipeline configurations, TTX and RP overheads (accounting for the error bars) show weak scaling performance

When the workload includes the EGFR kinase, we see (Fig. 6) that the RP overhead becomes on average less than 10% of the average total execution time (TTX), defined as *TTX*=*TTC*−*T*_*q*_ where *TTC* is time-to-completion and *T*_*q*_ is time spent queuing on the HPC machine. We further break down TTX into the time-to-completion per stage, where stages 1,2, and 7 perform file movements, while stages 3,4,5, and 6 execute NAMD tasks. At this level, we notice that the time-to-completion of the NAMD stages are essentially invariant across pipelines of different size while file movement stages exhibit linearly increasing behavior. In addition, when accounting for variance, RP overheads also show linear weak scaling behavior. As expected, EnTK overhead remains superlinear and comparable to the one measured in Experiment 1. This is because in both experiments EnTK overhead depends on the number of tasks translated to CU descriptions.

## Discussion

We designed experiments in Table 1 to characterize the overheads of the ESMACS protocol with null and NAMD workloads, respectively. Experiments 1 and 2 show how the overheads of both EnTK and RP tend to be invariant across type of workload executed. Their scaling behavior and, to some approximation, their absolute values are comparable between Figs. 5 and 6. This is relevant because it shows that the physical systems used to coordinate and execute the ESMACS protocol add a constant and comparatively not relevant overhead to the execution of NAMD.

## Conclusion

It is necessary to move beyond the prevailing paradigm of running individual MD simulations, which provide irreproducible results and cannot provide meaningful error bars [22]. Further, the ability to flexibly scale and adapt ensemble-based protocols to the systems of interest is vital to produce reliable and accurate results on timescales which make it viable to influence real world decision making. To meet these goals, we are designing and developing the high-throughput binding affinity calculator (HTBAC).

HTBAC employs the RADICAL-Cybertools to build ensemble-based applications for executing protocols like ESMACS at scale. We show how the implementation of the ESMACS protocol scales almost perfectly to hundreds of concurrent pipelines of binding affinity calculations on Blue Waters. This permits a time-to-solution that is essentially invariant of the size of candidate ligands, as well as the type and number of protocols concurrently employed.

The use of software implementing well-defined abstractions like that of “building blocks”, future proofs users of HTBAC to evolving hardware platforms, while providing immediate benefits of scale and support for a range of different application workflows. Thus, HTBAC represents an important advance towards the use of molecular dynamics based free energy calculations to the point where they can produce actionable results both in the clinic and industrial drug discovery.

In the short term, the development of HTBAC will allow a significant increase in the size of study. Much of the literature on MD-based free calculations is limited to a few tens of systems, usually of similar drugs bound to the same protein target. By facilitating the investigation of much larger set of systems, HTBAC contributes to solve the grand challenge in drug design and precision medicine: understanding the influences on binding strength for hundreds or thousands of drug-protein variant combinations. While contributing to reach this ambitious goal, we reveal the limits of existing simulation technology and of the potentials used to approximate the chemistry of the real systems.

## Abbreviations

API: Application programming interface
CU: Compute units
DAG: Directed acyclic graph
DB: Database
EGFR: Epidermal growth factor receptor
EnTK: Ensemble toolkit
ESMACS: Enhanced sampling with approximation of continuum solvent
HPC: High-performance computing
HTBAC: Targeted kinase inhibitors
HTC: High-throughput computing
LRMS: Local resource management system
MD: Molecular dynamics
MMPBSA: Molecular mechanics poisson-boltzmann surface area
MPI: Message-passing interface
NAMD: Molecular dynamics engine
NPT: Constant number (N) pressure (P) and temperature (T)
NVT: Constant number (N) volume (V) and temperature (T)
RCT: RADICAL-cybertools
RP: RADICAL-pilot
TI: Thermodynamic integration
TIES: Thermodynamic integration with enhanced sampling
TKI: Targeted kinase inhibitors
TQ: Queueing time
TTC: Time to completion
TTX: Total execution time
